# Less invasive beractant administration in preterm infants: a pilot study

**DOI:** 10.6061/clinics/2016(03)02

**Published:** 2016-03

**Authors:** Cristina Ramos-Navarro, Manuel Sánchez-Luna, Susana Zeballos-Sarrato, Noelia González-Pacheco

**Affiliations:** Complutense University, Gregorio Marañon University Hospital, Biomedical Research Institute Gregorio Marañon, Neonatology Division, Madrid/, Spain

**Keywords:** Beractant, Feasibility Studies, Infant, Premature, Non-Invasive Ventilation

## Abstract

**OBJECTIVES:**

The aims of this study were to assess the efficacy and feasibility of a new, less invasive surfactant administration technique for beractant replacement using a specifically designed cannula in preterm infants born at <32 weeks of gestation and to compare short- and long-term outcomes between this approach and standard treatment, consisting of intubation, administration of surfactant and early extubation to nasal continuous positive airway pressure.

**METHOD:**

This was a single-center, prospective, open-label, non-randomized, controlled pilot study with an experimental cohort of 30 patients treated with less invasive surfactant administration and a retrospective control group comprising the 30 patients most recently treated with the standard approach. Beractant (4 ml/kg) was administered as an exogenous surfactant in both groups if patients on nasal continuous positive airway pressure during the first three days of life were in need of more than 30% F_i_O_2_. Clinicaltrials.gov: NCT02611284.

**RESULTS:**

In the group with less invasive surfactant administration, beractant was successfully administered in all patients. Thirteen patients (43.3%) in the group with less invasive surfactant administration required invasive mechanical ventilation for more than 1 hour during the first 3 days of life, compared with 22 (73%) in the control group (*p*<0.036). The rate of requiring invasive mechanical ventilation for more than 48 hours was similar between the infants in the two groups (46% *vs*. 40%, respectively). There were no differences in other outcomes.

**CONCLUSION:**

The administration of beractant (4 ml/kg) using a less invasive surfactant administration technique with a specifically designed cannula for administration is feasible. Moreover, early invasive mechanical ventilation exposure is significantly reduced by this method compared with the strategy involving intubation, surfactant administration and early extubation.

## INTRODUCTION

Over the last few years, there has been a trend of less invasive respiratory management among preterm infants due to the association between invasive mechanical ventilation (iMV) and bronchopulmonary dysplasia (BPD) development. Currently, applying nasal continuous positive airway pressure (nCPAP) from birth combined with early surfactant administration to avoid iMV is a standard of care in preterm infants 1,2.

Until recently, exogenous surfactant administration has required intubation and positive pressure ventilation (PPV) during instillation. This implies that certain infants managed with nCPAP have had to be intubated only for the purpose of administering exogenous surfactant. Early surfactant treatment improves respiratory outcomes in patients with respiratory distress syndrome (RDS) 3, but as endotracheal intubation can be hazardous 4,5 and iMV exposure can induce lung injuries 6, the potential benefit of surfactant treatment is counterbalanced by ventilator-induced damage. To reduce iMV exposure, early extubation after surfactant administration (intubation-surfactant-extubation, or INSURE) has been proposed, but iMV is still required during surfactant administration and in certain cases (7-19%), extubation cannot be performed after treatment 7,8. To prevent endotracheal intubation and iMV during and after surfactant administration, the decision to administer exogenous surfactant can occasionally be delayed.

To prevent intubation but ensure early surfactant administration, several techniques for “less invasive surfactant administration” (LISA) have been described recently. In these techniques, the exogenous surfactant can be delivered without tracheal intubation, instead using nasopharyngeal instillation 9, laryngeal mask placement 10 or aerosolization 11. However, none of these methods is currently ready for application. Another more widely used method is to use a thin catheter while the infant breathes spontaneously, supported with nCPAP. A thin (5- to 6-gauge) feeding tube has been used, with the disadvantage of needing to use a Magill forceps for its placement 12,13,14,15.

To address this problem, certain researchers have modified the technique by using a more stable vascular catheter 16-18. This method has been analyzed in comparative studies. A recent review that included 2,361 treated neonates 19 concluded that exogenous surfactant administration via a thin catheter might be an efficacious and safe method, with a potential reduction in the need for mechanical ventilation within the first 72 hours of birth compared with standard care. In the present study, to reduce invasiveness and ensure security, we tested a specifically designed surfactant replacement tube that is rigid enough to be placed in the trachea without the need to employ any forceps and that has a blunt tip to avoid any possible damage during placement. Beractant (Survanta®) is a natural surfactant not documented for use in the LISA technique; the high-volume dose needed (4 ml/kg) is probably a concern limiting its use. The particular aims of this study were to assess the feasibility and effectiveness of 100 mg/kg (4 ml/kg) beractant administered by LISA using a specifically designed surfactant replacement tube and to compare the short- and long-term respiratory outcomes between patients treated with this approach and a cohort of similar historical controls.

## MATERIALS AND METHODS

This single-center, prospective, open-label, non-randomized, historically controlled pilot study involved an experimental cohort of 30 patients treated with a new LISA technique and a retrospective control group comprising the 30 patients most recently treated with surfactant using the standard method who met the inclusion criteria. Preterm infants born at <32 weeks of gestation (WG) and breathing spontaneously on nCPAP during the first three days of life who met the exogenous surfactant administration criteria (Table 1) were eligible to enroll in the study. Infants who met the intubation criteria (Table 1) at the moment of surfactant administration were excluded from both groups.

### Treatment cohort (LISA)

**Figure 1-:**
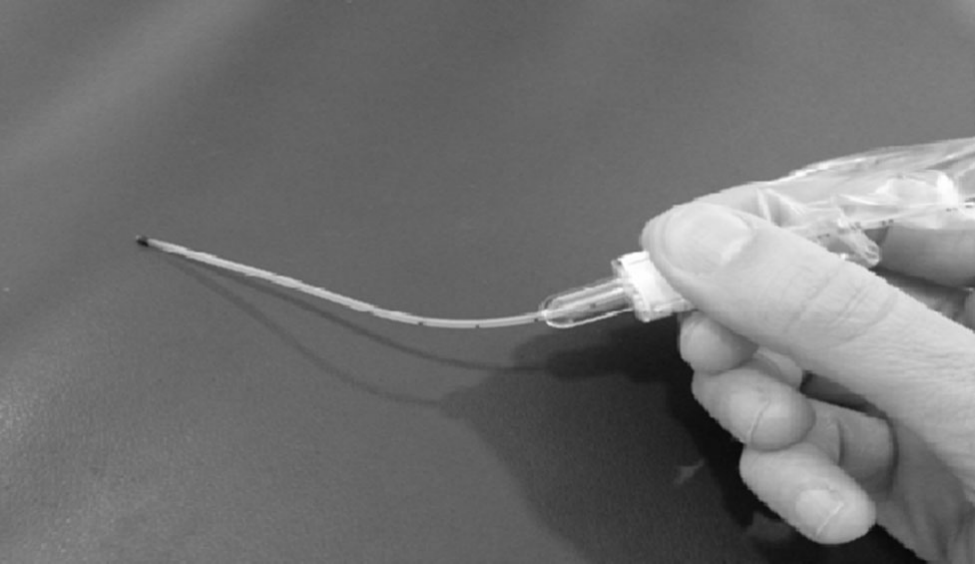
A 5-gauge French, gamma-sterilized, multi-access catheter specifically designed to deliver surfactants in neonates/pediatric patients.

All preterm infants born at <32 WG between October 2013 and November 2014 who met the inclusion criteria were managed using the new LISA technique and enrolled in the study group. All infants were supported with nCPAP (5-8 cmH_2_O) while breathing spontaneously during instillation, delivered by an Infant Flow Driver® device (Care Fusion, San Diego, CA, USA) via short bi-nasal prongs. All preterm infants were properly positioned by a second attending person, who contained the infant and facilitated tucking during the procedure. The use of oral sucrose or breast milk 2 minutes before the procedure was encouraged to minimize the need for sedatives and opioids, but this use was allowed at the discretion of the attending neonatologist. A 5-gauge French, gamma-sterilized, multi-access catheter specifically designed (Figure 1) to deliver surfactants in neonates/pediatric patients (KimVent Trach Care Technology®, Kimberly-Clark Health Care, West Malling, Kent, United Kingdom) was placed 1-2 cm below the vocal cords by direct laryngoscopy, without the use of a Magill forceps. Correct positioning of the catheter tip was ensured based on the numeric marks on the catheter. Beractant was used as the exogenous natural surfactant, (100 mg/kg; 4 ml/kg) and was administered in two aliquots over 1-3 minutes. All infants were monitored by continuous electrocardiogram (EKG) and peripheral pulse oximetry. Positive pressure inflation (PPI) was given using a mask and bag only if the infant was apneic or if bradycardia developed, despite the interruption of the procedure. During surfactant instillation, gastric aspiration was performed through a standard nasogastric feeding tube to discount any misplacement of the surfactant catheter. The surfactant administration catheter was removed after surfactant instillation and nCPAP support was maintained, with F_i_O_2_ adjusted for 90-95% target SpO_2_. A second dose of surfactant was administered during the first 3 days of life if more than 40% F_i_O_2_ was needed while on nCPAP with at least 6 cmH_2_O pressure. All infants received prophylactic intravenous caffeine citrate (loading dose of 20 mg/kg, followed by 5 mg/kg/d) during the first 8 hours of life. LISA failure was considered when intubation during the first three days of life was required.

### Historical cohort (standard treatment)

The control group was collected from the period immediately before the study's initiation (from June 2012 to September 2013). This cohort comprised preterm infants born at <32 WG who met the inclusion criteria. Standard management was performed using beractant (4 ml/kg) after endotracheal intubation. A multi-access catheter designed to deliver surfactants in neonates/pediatric patients (KimVent Trach Care Technology®, Kimberly-Clark Health Care, West Malling, Kent, United Kingdom) was pre-connected to the endotracheal tube and used for surfactant administration without having to disconnect the ventilator. While surfactant was administered, all infants were connected to pressure support ventilation (PSV) combined with volume guarantee (VG), Dräger VN500 ventilator (Dräger Medical, Lübeck, Germany). A tidal volume of 4 ml/kg was initially employed.

After surfactant administration, all infants were to be extubated, in accordance with our institutional extubation guidelines, if F_i_O_2_<0.35, for a target SpO_2_ of >90%, and if a consistent respiratory effort was present. Extubation was supported with nCPAP in all patients.

### Outcomes

The primary endpoint was the percentage of patients who required more than 1 hour of mechanical ventilation during the first three days of life. Secondary endpoints were the need for iMV at any time, its duration and the requirement of a second dose of surfactant. Other outcomes collected were the incidence of patent ductus arteriosus (PDA), BPD (moderate to severe according to the physiological definition 17) or air leaks.

To assess the feasibility of the technique, the number of attempts to catheterize the trachea was recorded, as was the number of bradycardia episodes (<100 bpm) needing PPI during instillation, the number of surfactant reflux cases and any misplacement of the catheter. The effectiveness of surfactant administration was assessed in both groups based on reduction of the F_i_O_2_ requirement by more than 20%.

### Statistical analysis

A convenience sample size of 30 patients in each group was selected for this pilot study.

Variables of interest are expressed as percentages, as means (standard deviations) for normally distributed continuous variables and as medians (ranges) for non-normally distributed variables according to the Kolmogorov-Smirnov test. Fisher's exact test (two-tailed) or the Mann-Whitney's U test was used to establish baseline differences between the infants in the LISA and control cohorts. Additionally, logistic regression analysis was used to investigate the association between the LISA and standard treatment procedures and the need for mechanical ventilation. These analyses were adjusted for the effects of gestational age (GA) and the Critical Risk Index for Babies (CRIB) score. Selected clinical variables were compared between LISA success and LISA failure through a univariate comparison. A *p* value of <0.05 was considered statistically significant. The data were analyzed using SPSS package version 19.0 (SPSS, Chicago, IL, USA) for Windows.

## ETHICS

The Institutional Ethics Committee approved the study and written consent was also obtained for the prospective LISA cohort.

## RESULTS

From October 2013 to November 2014, 98 preterm infants were born at <32 WG at our level III institution. Overall, 65.3% of infants (n=64) could be managed without intubation at birth. A total of 23 of the patients on nCPAP from birth met the surfactant administration criteria and were included in the study group. In addition, seven patients who were intubated in the delivery room for stabilization and extubated after surfactant treatment met the inclusion criteria for their second dose of surfactant, so a total of 30 patients were included in the study group.

From June 2012 to September 2013 (the control period), 148 infants were born at <32 WG, and 96 (64.8%) did not need intubation at birth. Of these 96 spontaneously breathing patients supported with nCPAP, 35 met the inclusion criteria. We selected the 30 patients born immediately before to form the control group.

There were no differences between the two groups in terms of baseline characteristics or prenatal risk factors, except for the Clinical Risk Index for Babies (CRIB) Score, which was significantly higher in the LISA group, indicating higher initial severity in clinical status (Table 2).

Beractant (4 ml/kg) was administered in both groups.

The LISA technique was feasible in all of the patients and was executed by a senior neonatologist in 23 patients and by a neonatology fellow in 7 patients. The catheter was properly inserted in all patients and the surfactant was administered without any significant incident. None of the patients in the LISA group required intubation for surfactant administration. For all patients who did need intubation, this was performed more than 1 hour after the procedure. The surfactant was specifically administered in two divided aliquots over 1-2 minutes and all infants exhibited hemodynamic stability during and after the procedure. A F_i_O_2_ reduction of more than 20% during the first hour after surfactant administration was observed in 73.3% of the LISA group, compared with 86.6% in the control group (*p*>0.05). Slight reflux of the surfactant was observed in 2 patients (6.7%) in the LISA group, and an adequate response to surfactant was noted in both groups. No sedatives were given in the LISA group. In the control group, analgesics (morphine or fentanyl) were administered in 9 infants (30%). The failure rate was 77.7% in the control group in which analgesics were administered compared with 62% in the control group in which analgesics were not used (*p*>0.05).

### Primary outcome

Thirteen patients (43.3%) in the LISA group required iMV for more than one hour during the first three days of life, compared with 22 (73%) in the standard treatment control group (OR: 3.596; 95% CI 1.216-10.638; *p*=0.02). After adjustment for GA and the CRIB score, the benefit of the LISA technique was even more evident (OR 6.484; 95% CI 1.689-24.893; *p*=0.006).

### Secondary outcomes

**Figure 2-:**
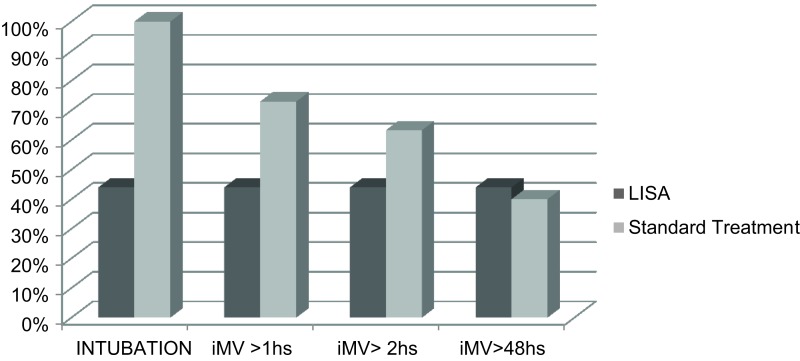
Patients who needed intubation and invasive mechanical ventilation after less invasive surfactant administration or standard treatment.

Secondary outcomes are shown in Table 3. There were no statistically significant differences in the need for a second dose of surfactant, the duration of mechanical ventilation, or the PDA or BPD incidence rate. The rate requiring iMV for more than 48 hours was similar between the infants in the two groups (46% *vs*. 40%, respectively) (Figure 2). The risk factors associated with LISA failure were a lower GA and a lack of reduction in F_i_O_2_ after surfactant administration (RR: 3.208; 95% CI 1.545-6.664; *p*=0.012) (Table 4). Patients with LISA failure had longer endotracheal mechanical ventilation than the group with LISA success did (median number of days: 196.46 *vs*. 9.18, respectively; *p*=0.04).

## DISCUSSION

The LISA technique using a specifically designed catheter for 4 ml/kg beractant administration is feasible and safe and decreases early iMV exposure in comparison with standard treatment. To our knowledge, this is the first study of a LISA technique using beractant as the exogenous surfactant and a specifically designed catheter for surfactant administration.

Currently, avoidance of intubation is one of the main targets in respiratory management among preterm infants, especially in the first few hours of life, due to the association between ventilator-induced lung injury and BPD 6. In addition, early surfactant administration improves respiratory outcomes compared with later use in patients with RDS 1,20. The decision to administer surfactant in a patient with spontaneous breathing is difficult and is occasionally delayed to avoid intubation and invasive ventilation through the endotracheal tube. Even with the INSURE method, a brief period of PPV is required and at times, extubation cannot be rapidly performed 7,8. In the present study, 73% of infants were exposed to iMV for more than 1 hour after surfactant treatment in the control group. Possible reasons for prolonged ventilation were the use of analgesic with impairment in respiratory drive and a high ventilator requirement after surfactant administration.

A current controversy surrounding this issue relates to the use of sedatives and analgesics. One of the main reasons for INSURE failure in many reports is apnea or insufficient respiratory drive related to sedative use in many cases 21. In fact, there is insufficient safety evidence to recommend a drug and dose to be used in preterm infants during the intubation procedure, particularly if the respiratory drive must be preserved 22,23. The AMV trial 19 evidenced a high risk of non-invasive surfactant failure in those infants in whom sedation was administered (60% *vs*. 22%).

The use of pharmacological analgesics was allowed in both groups in the present study, at the recommendation of the attending neonatologist. However, for LISA treatment, specific non-pharmacological measures were established to reduce the need for sedatives. Major concern about the need for a preserved respiratory drive, the positive effect of contention and sucrose or breast milk 22 and the performance of this technique by a senior neonatologist probably contributed to the lack of use of sedatives in the LISA group, in contrast to the 30% analgesic use in the control group. Even though apnea due to analgesic use can contribute to an increased rate of iMV after surfactant administration, we did not find significant differences in the rate of iMV over more than 1 hour in the control group in which analgesics were administered compared with the control group in which analgesics were not used (77.7% *vs*. 62%, respectively; *p*>0.05). The LISA technique was performed in only one attempt in 83.3% of cases. We believe that by simplifying the technique, it can be performed easily, with a very short laryngoscopy time; as a result, the use of analgesics or sedation will not be necessary. Properly preventing sedative use ensures the respiratory drive and also avoids the drop in blood pressure and impairment of cerebral perfusion that have been observed in preterm infants after sedative use 20,21,22. This lack of analgesic use has been reported in several previously published trials of LISA techniques 18,.

The use of a specifically designed tube for surfactant administration, one that is rigid enough to prevent the use of forceps but that has a soft tip to prevent cord damage, made the technique presented here easy and safe. Despite the increased amount of beractant administered (4 ml/kg) compared with other surfactant concentrations, it was well tolerated and patients did not experience adverse effects. The rates of surfactant reflux and bradycardia during instillation (6.7% and 10%, respectively) in this study were similar to those in other reports (20-40%) 16,19,23. However, a slight drop (>100 bpm) in heart rate was more frequently observed (30%), which was resolved by transient interruption of the procedure until resolution. To our knowledge, no other studies have examined the use of beractant via a LISA technique. The AMV trial 19 included 14 patients in whom beractant was administered by a less invasive technique, but no specific details of the procedure were reported.

Regarding surfactant administration effectiveness (more than 20% F_i_O_2_ reduction), no differences were found between the groups (*p*=0.08). All infants not responding to surfactant administration by LISA required iMV. In this group, a second dose of surfactant was administered in 62.2% of cases (n=5), with one administered by LISA and the others administered after intubation. Once again, F_i_O_2_ reduction was not observed. The total rate of requiring a second dose of surfactant was similar between the infants in the two groups (33.3% in the LISA group and 30% in the control group). This finding was in contrast to the observation by Aguar et al. 24 that the non-invasive surfactant group received a second dose of surfactant at a significantly higher frequency compared with the INSURE group. This fact supported the researchers' hypothesis that attributes intergroup differences to a higher dose of surfactant applied in the INSURE group (200 mg/kg) compared with the less invasive group (100 mg/kg), rather than being caused by the technique itself. In contrast, in the present study, 100 mg/kg was administered in both groups. A second dose was administered by the LISA technique in 3 patients, one of whom did not require iMV. Patients who needed intubation after LISA required prolonged ventilation, with a duration of more than 48 hours in all cases. This rate (46%) was similar to that for the standard method (40%) (Figure 2). No significant differences between the groups were found for the other outcomes (Table 3). It seems that by changing the technique, we can avoid unnecessary early intubations for surfactant treatment but that more severe ill infants will still require mechanical ventilation.

The benefit of surfactant administration by this technique is the avoidance of the use of unnecessary iMV around surfactant administration, which can trigger an inflammatory response in the lung and systemic circulation 6,27. This systemic repercussion could explain the positive effect of LISA on survival, without major complications, found in a recently published randomized controlled trial by Kribs et al. 28. Additionally, iMV during surfactant administration has been shown to reduce the effect of the surfactant administered 29,30, contributing to impaired respiratory evolution. The LISA technique allows early surfactant administration without the fear of unnecessary iMV exposure and spontaneous breathing during LISA may contribute to a better distribution of surfactant.

In the present study, no significant difference was found in the death or BPD (II-III) rate between the LISA group (26.6%) and the control group (30%) (Table 3). A recent review of 2,630 patients found no statistically significant reduction in BPD with surfactant administration via a thin catheter when compared with INSURE or standard care 16,30.

In our study, all BPD patients in the LISA group had moderate BPD (type II). There were 2 infants with severe BPD in the control group. Even though no difference in oxygen dependency was found at 36 WG, long-term follow-up with an appropriate sample size is needed to assess any possible beneficial effects of avoiding early intubations using this technique on long-term lung function.

Based on the variables included in the univariate comparison between the LISA success and failure groups, the risk factors associated with LISA failure were a lower GA and a lack of reduction in F_i_O_2_ (Table 4).

### Limitations

In this feasibility pilot study, the prospective study group was compared with a historical control group. There was temporal bias caused by the evolution of medical practice and technology, although this bias was reduced because the control group consisted of immediately before born preterm infants that met the same surfactant administration criteria as LISA group. The reasons for prolonged iMV after standard treatment could not be properly collected in certain patients. Moreover, due to the low number of patients included, the study was not powered to examine the BPD outcome.

The LISA technique using a KimVent catheter for 4 ml/kg beractant administration is feasible and safe and reduces iMV exposure in the first 3 days of life compared with the previously established standard method. There are no differences in other outcomes. The risk factors associated with LISA failure were a lower GA and a lack of reduction in FiO2 after surfactant administration. LISA failure indicates more severe RDS that requires prolonged mechanical ventilation.

## ACKNOWLEDGMENTS

The authors would like to thank Adriana López-Pineda for assistance in drafting and editing the manuscript.

## AUTHOR CONTRIBUTIONS

Ramos-Navarro C and Sánchez-Luna M participated in the study conception and design, the analysis and interpretation of the data, drafting and revision of the manuscript as submitted. Zeballos-Sarrato S participated in the study conception and the revision of the manuscript as submitted. González-Pacheco N participated in the analysis and interpretation of the data of the manuscript as submitted.

## ACKNOWLEDGMENTS

The authors would like to thank Adriana López-Pineda for assistance in drafting and editing the manuscript.

## Figures and Tables

**Table 1:** Surfactant administration and intubation criteria in preterm infants on nCPAP PEEP ≥ 6cmH2O) (control and less invasive surfactant administration groups).

Surfactant Administration Criteria	Intubation Criteria
	F_i_O_2_>50% (SpO_2_ 90-95%)
	First dose: F_i_O_2_>30% (SpO_2_ 90-95%) Second dose: F_i_O_2_>40% (SpO_2_ 90-95%)	Apnea episodes (>4/h or more than 1 requiring PPV)
		Respiratory acidosis, pCO_2_>65 mmHg and pH<7.20 in arterial or capillary samples

PEEP: Positive end-expiratory pressure.

**Table 2:** Baseline characteristics of the study population.

	LISA N=30	STANDARD TREATMENT N=30	*p* (95% CI)
Gestational age (weeks), mean	28.4	29.1	0.15
N≤29 weeks	17	14	
N>29 weeks	13	16	
Birth weight (grams), mean	1058	1232	0.26
Male gender, n(%)	12(40)	18(60)	0.12
Prenatal steroids, n(%)	22(73)	21(70)	0.42
C-section, n(%)	24(80)	22(73.3)	0.54
Intubation at delivery, n(%)	7(23.3)	0	0.05
CRIB score, mean;SD	4;3.08	1.9;2.09	0.003
Age at procedure (hours), mean	11.4	11	0.76
First dose of surfactant, n(%)	23(76.7)	30(100)	0.005
F_i_O_2_ prior to surfactant administration, %	42	40	0.59

C-section: Caesarean section.

**Table 3:** Postnatal respiratory management of less invasive surfactant administration and INSURE groups.

	LISA N=30	STANDARD TREATMENT N=30	*p* (95% CI)
Gestational age (weeks), median	28.4	29.1	0.15
Age at procedure (hours), mean(SD)	11.4(14.7)	11(14.7)	0.76
2 or more attempts, n(%)	5(16.7)	-	
Bradycardia (>10 seg), n(%)	3(10)	-	
Surfactant reflux, n(%)	2(6.7)	-	
F_i_O_2_ before procedure, %	42	40	0.58
F_i_O_2_ reduction (>20%), n(%)	22(73.3)	26(86.6)	0.08
Second dose of surfactant, n(%)	10(33.3)	9(30)	0.39
Pneumothorax, n(%)	2(6.7)*	1(3.3)	0.55
iMV >1 hour during first 3 days, n(%)	13(43)	22(73)	**0.036**
Total iMV (hours)	84.12	82.22	0.37
PDA, n(%)	11(36.7)	12(40)	0.79
Death or BPD (II-III), n(%)	8(26.6)	9(30)	0.61
Pharmacological analgesics, n(%)	0(0%)	9(30%)	<0.05

**<?ENTCHAR ast?>:** previously.

LISA: less invasive surfactant administration.

BPD: bronchopulmonary dysplasia; iMV: invasive mechanical ventilation; PDA: patent ductus arteriosus; PPI: positive pressure inflations; SD: standard deviation.

**Table 4:** Comparison of variables between the less invasive surfactant administration success and failure groups.

	LISA Failure N=13	LISA Success N=17	*p* (95% CI)
Gestational age (weeks), mean(SD)	27.4(1.61)	29.1(2.02)	**0.02**
Birth weight (grams), mean(SD)	992(387)	1108 (397)	0.263
Prenatal corticosteroids, n(%)	9(69.2)	13(76.5)	0.691
Intubation at delivery, n(%)	30.8	17.6	0.4
Age at procedure (hours), mean(SD)	8.4(9.6)	13.7(17.7)	0.7
F_i_O_2_ before procedure, mean(SD)	4.6(37.6)	21.6(46)	0.5
F_i_O_2_ reduction after surfactant (>20%), n(%)	6(46.2)	16(94.1)	**0.003**
PPI during instillation, n(%)	2(15.4)	1(5.9)	0.39
PDA, %	46	29.4	0.3
Total iMV (hours), mean(SD)	196.46(244)	9.18(18.7)	**0.04**
Pneumothorax, n(%)	2(15.4)	0	0.09
BPD (II-III), %	30.8	25.6	0.907

LISA: less invasive surfactant administration.

BPD: bronchopulmonary dysplasia; iMV: invasive mechanical ventilation; PPI: positive pressure inflations; SD: standard deviation.
